# Impact of vaccination against COVID-19 on patients with cancer in ACHOC-C19 study: Real world evidence from one Latin American country

**DOI:** 10.7150/jca.79969

**Published:** 2023-07-31

**Authors:** Aylen Vanessa Ospina, Ricardo Brugés, Iván Triana, Guillermo Sánchez-Vanegas, Angela Barrero, William Mantilla, Pedro Ramos, Laura Bernal, Sandra Aruachán, Manuel González, José Lobatón, Alicia Quiroga, Giovanna Rivas, Guido González, Milton Lombana, Isabel Munevar, Paola Jiménez, Ana Cristina Avendaño, Mirian Caicedo Arias, Carolina López, Héctor González, Javier Pacheco, Ray Manneh, Paola Pinilla, Andrea Russi, Juan Ortiz, Jesús Insuasty, Carmen Alcalá, Fernando Contreras, Juliana Bogoya

**Affiliations:** 1 ICCAL Fundación Santa Fe de Bogotá. Asociación Colombiana de Hematología y Oncología - ACHO.; 2 Instituto Nacional de Cancerología - Pontificia Universidad Javeriana.; 3 Hospital Universitario Mayor - Mederí. Universidad del Rosario.; 4 Asociación Colombiana de Hematología y Oncología - ACHO. Instituto Nacional Cancerología.; 5 Fundación Cardio infantil, Universidad del Rosario, Grupo ICAROS.; 6 Clínica Universitaria Colombia Sanitas.; 7 Clínica Universitaria Colombia Sanitas - Clínica Marly.; 8 IMAT Oncomédica.; 9 Hospital Pablo Tobón Uribe.; 10 Centro Médico Clínica de Occidente.; 11 Hospital Militar Central, Fundación Cardioinfantil. Hemato Oncólogos Asociados.; 12 Hospital Militar Central, Hemato Oncólogos Asociados.; 13 Hemato Oncólogos Asociados, Instituto de Oncología.; 14 CECANCOL.; 15 Hospital de San José.; 16 Sociedad de Oncología y Hematología del Cesar.; 17 Hospital Universitario San Ignacio.; 18 Hospital Universitario del Valle.; 19 Hospital Universitario de Santander - Universidad Industrial de Santander.; 20 Clínica de la Costa.; 21 Instituto Nacional Cancerología. Universidad El Bosque.; 22 Instituto Nacional Cancerología.

**Keywords:** COVID-19, vaccines, cancer, vaccine effectiveness, cohort studies, mortality, mechanical ventilation, hospitalization

## Abstract

**Introduction:** During the pandemic, it has been recommended that vaccination against COVID-19 be a priority for patients with cancer; however, these patients were not included in the initial studies evaluating the available vaccines.

**Objective:** To define the impact of vaccination against COVID-19 in preventing the risk of complications associated with the infection in a cohort of patients with cancer in Colombia.

**Methods:** An analytical observational cohort study, based on national registry of patients with cancer and COVID 19 infection ACHOC-C19, was done. The data was collected from June 2021, until October 2021. Inclusion criteria were: Patients older than 18 years with cancer diagnosis and confirmed COVID-19 infection. Data from the unvaccinated and vaccinated cohorts were compared. Outcomes evaluated included all-cause mortality within 30 days of COVID-19 diagnosis, hospitalization, and need for mechanical ventilation. The estimation of the effect was made through the relative risk (RR), the absolute risk reduction (ARR) and the number needed to treat (NNT). Multivariate analysis was performed using generalized linear models.

**Results**: 896 patients were included, of whom 470 were older than 60 years (52.4%) and 59% were women (n=530). 172 patients were recruited in the vaccinated cohort and 724 in the non-vaccinated cohort (ratio: 1 to 4.2). The cumulative incidence of clinical outcomes among the unvaccinated vs vaccinated patients were: for hospitalization 42% (95% CI: 38.7%-46.1%) vs 29%; (95% CI: 22.4%-36.5%); for invasive mechanical ventilation requirement 8.4% (n=61) vs 4.6% (n=8) and for mortality from all causes 17% (n=123) vs 4.65% (n=8).

**Conclusion:** In our population, unvaccinated patients with cancer have an increased risk of complications for COVID -19 infection, as hospitalization, mechanical ventilation, and mortality. It is highly recommended to actively promote the vaccination among this population.

## Introduction

As of February 2022, more than 6,000,000 COVID-19 cases have been confirmed in Colombia, and around 139,000 people have died from this disease. In the literature, it has been described that patient with cancer, especially those with metastatic disease, are a group of interest due to their high risk of associated complications and high incidence of mortality 1. According to local data from our ACHOCC-19 study, 30-day mortality was observed in 26.3% 2 of the patients.

To counter the effect of the pandemic, one of the strategies implemented was the rapid development of vaccines for COVID-19 3, whose worldwide distribution began at the end of 2020. Patients with cancer were not included in clinical studies developed for the evaluation and approval of vaccines. However, given the risk factors of this population, their use was systematically recommended by all academic and scientific associations such as ASCO (American Association of Clinical Oncology) 4 and ESMO (European Association of Medical Oncology) 5. Additionally, these same associations have encouraged collecting data on the results and outcomes of vaccination in cancer patients, given the lack of information.

In that sense, during the last two years, information has been obtained from multiple studies worldwide on the efficacy and safety of vaccination against COVID-19 in patients with cancer. These reports have also provided evidence to identify the subgroups at higher risk and to propose the need for boosters, given the duration of immunity in this population. However, the data available on the efficacy and safety of vaccination in the Latin American population is limited 6-8.

On February 17th, 2021, the first doses of the vaccine against COVID-19 were administered in Colombia, initially to health personnel and progressively prioritized the population, according to risk factors, until reaching cancer patients. for whom vaccination was available as of June 2021. Specifically, patients in Colombia have had access to different alternatives: the chemically inactivated vaccine, CoronaVac, designed to be administered in two doses and with a seroconversion rate greater than 90% 9; Two vaccines with viral vector technology, ChAdOx1 nCoV-19, with a two-dose schedule and reported efficacy between 62 and 90% 10; Ad26.COV2.S, proposed for an initial one-dose schedule, with a reported percentage of seroconversion from 80 to 100% 8; And finally, two vaccines with nucleic acid technology encoding a protein antigen, BNT162b2 12, 13, and mRNA-1273 14, 15, both to be administered initially in two-dose regimens and with reported seroconversion percentages of 100%.

Even though different medical associations worldwide, including the European Society of Medical Oncology and the American Society of Clinical Oncology, have recommended that vaccines against COVID-19 be a priority for cancer patients 16, in countries like Colombia, adequate vaccination rates for this high-risk population have not been achieved during first few months of vaccine availability. This situation could be due, in part, to the dissemination of unverified information, which increases the fear and doubts of patients and their families, without ruling out problems of coverage and heterogeneity in access to the health system. Additionally, there are no national data on the efficacy of vaccination in patients with cancer.

The Asociacion Colombiana de Hematologia y Oncologia (ACHO), aware of this problem, has been working since the beginning of the pandemic, on generating local scientific evidence. For this reason, the present research was developed to establish the effectiveness of COVID-19 vaccines in cancer patients who have had a confirmed SARS-CoV2 infection, based on a comparison of the incidence of hospitalization, mechanical ventilation, and 30-day all-cause mortality from the cohort of unvaccinated and vaccinated patients.

## Materials and methods

***Design, population and sample:*** An observational analytical cohort study was carried out based on data from COVID-19 infection in patients with cancer ACHO national registry (ACHOC-C19). This research included the information of patients over 18 years of age with a confirmed diagnosis of cancer (solid tumors) and a confirmed COVID-19 infection. The data of the exposed cohort (unvaccinated) and the unexposed cohort (vaccinated) were compared, starting in June 2021, when the vaccination process of cancer patients in Colombia began, with a cutoff date of October 30th of the same year. The registry data comes from the care of a group of 37 oncologists located in the country's main cities.

The sample size was calculated using the arcsine method 17, taking into account the following assumptions: a RR of 0.41 (95% CI: 0.22-0.77) for the outcome of all-cause mortality in vaccinated patients, according to the results of the systematic review published by Korang et al. 18; an incidence of mortality in patients with cancer and COVID-19 infection in Colombia of 26%, according to the results of Ospina et al.; an alpha value of 5%, a type II error of 20% and a ratio of 1 to 4 between vaccinated and unvaccinated patients. Based on the above, a minimum required sample size of 84 vaccinated patients and 336 unvaccinated patients was estimated for a total of 420 subjects.

***Enrollment, monitoring and data processing procedure:*** The ACHOC-C19 registry is based on the contribution of a group of medical oncologists and institutions responsible for caring for cancer patients in Colombia. Each patient who met the inclusion criteria was registered in the data capture system, in which a series of sociodemographic and clinical variables were documented. The moment of admission to the cohort was given by the event of infection by COVID-19 in a patient diagnosed with cancer. Only a patient with a complete vaccination schedule for COVID-19 was considered in the unexposed cohort, while unvaccinated individuals were included in the exposed cohort. In addition, each physician recorded the patient's follow-up for thirty days after the condition of COVID-19 infection.

***Variables of interest:*** In addition to the exposure variable (vaccination status of the patient), the following variables were included in the analysis: age, sex, place of residence (rural or urban), socio-economic level, type of health insurance scheme (contributory or subsidized), comorbidities such as diabetes, obesity, smoking, ECOG at admission to follow-up, TNM, type of tumor and current treatment.

The primary outcome was all-cause mortality 30 days after COVID-19 infection. Secondary outcomes included hospitalization for COVID-19 and the requirement for mechanical ventilation associated with COVID-19 infection.

***Statistical analysis:*** A descriptive analysis was carried out according to the measurement scale of the different variables. Absolute and relative frequency measures were used for qualitative variables, while numerical variables were described using measures of central tendency (mean and median) and dispersion (standard deviation and interquartile range). The cumulative incidence of the three events of interest was calculated for the unvaccinated and vaccinated cohorts. Based on these results, the relative risk (RR), the absolute risk reduction-ARR (risk difference) and the number needed to treat-NNT (1/ARR) were estimated. Each estimator was accompanied by its respective 95% confidence interval. The Chi-square test was applied to test the hypothesis of association or independence. The calculation of effect measures adjusted by covariates of interest or possible confounding factors was performed using generalized linear models of the binomial family. The selection of the models presented was based on criteria of clinical relevance and statistical adjustment (deviance, AIC, BIC). All analyzes were performed with the Stata 16 I/C statistical software.

### Ethical aspects

This study respected the ethical principles of the Declaration of Helsinki (2013) and the Resolution 8430 of 1993 of the Colombian Ministry of Health. The information was guaranteed to be for scientific purposes only, and the right to privacy was protected by the omission of the identification data of the study subjects. The protocol of this research was presented and approved by all the ethics committees of the participating entities.

## Results

A total of 896 patients were included, of which 470 were older than 60 years (52.4%), and 59% were women (n=530). The non-vaccinated cohort was made up of 724 patients, while the vaccinated cohort was made up of 172, distributed in CoronaVac (n=96; 55.8%), BNT162b2 (n=37; 21.5%), Ad26.COV2.S ( n=10, 5.8%), ChAdOx1 nCoV-19 (n=8, 4.6%), mRNA-1273 (n=4, 2.3%), and 17 patients who did not report the name of the vaccine (9.9%). All subjects were followed up 30 days after the onset of their clinical condition consistent with COVID-19 infection. In the comparison of baseline characteristics between the unvaccinated and vaccinated groups, significant differences were observed for the following factors (p-value <0.05): age over 60 years (63.95% vs. 49.86%), breast cancer (39.53 vs. 21.13), members of the subsidized health regime as surrogate of low socio-economic level (15.12 vs. 25.55) and in patients with metastatic disease/ TNM IV (23.26 vs. 33.29). Table 1 shows the details of the baseline characteristics of the entire sample and by vaccinated and non-vaccinated cohort.

### Effectiveness of vaccines against the risk of hospitalization

Of the 724 unvaccinated patients, 307 were hospitalized for their condition of COVID-19 infection, which translates into a cumulative hospitalization incidence of 42% (95% CI: 38.7%-46.1%). In the vaccinated cohort, 50 of the 172 patients were hospitalized that means incidence of hospitalization: 29%; (95% CI: 22.4%-36.5%). The crude RR was 1.45 (95% CI: 1.13-1.86), the ARR of 13% (95% CI: 5.6%-21%) and the NNT of 7.5 (95% CI: 4.7- 17.7). When adjusted for the variables age over 60 years, TNM-IV, subsidized health regimen/low socio-economic status, chemotherapy, and breast cancer, the RR was 1.36 (IC95%: 1.08-1.72), the ARR 12.5% (95% CI: 5.3%-19.6%) and the NNT 7.9 (95% CI: 5.1- 18.7). The detail of the multivariate analysis is presented in Table 2.

### Effectiveness of vaccines against the risk of mechanical ventilation

In the cohort of unvaccinated patients, 61 required mechanical ventilation during their hospitalization for COVID-19 (8.4%; 95% CI: 6.5%-10.7%); compared with the vaccinated cohort, where eight patients received this support, for a cumulative incidence of mechanical ventilation of 4.6% (95% CI: 2%-8.9%). The crude effect estimates were: RR 1.81 (95% CI: 0.9-3.7), ARR 3.8% (95% CI: 0.03%-7.5%) and NNT de 26.5 (IC95%:13.3-3049). After adjusting for the previously mentioned variables, the results of the effect were: RR 2.06 (95% CI: 1.01-4.24), ARR 4.5% (2.3%-6.7%) and NNT 22.1 (95% CI: 14.8-43.3). Table 2 presents the details of the model.

### Vaccine effectiveness against mortality risk

A total of 117 patients died in the unvaccinated patient cohort, for an incidence of 30-day all-cause mortality of 16.2% (95% CI: 13.5%-19.04%). Comparatively, 8 of the 172 patients died in the cohort of vaccinated patients, for a risk of 4.6% (2.02%-8.95%). The crude RR was 3.47 (95% CI: 1.73-6.97), the ARR 11.5% (95% CI: 7.4%-15.6%) and the NNT 8.7 (95% CI: 6.4- 13.6). In the multivariate analysis model, after adjusting for the predictors described, the RR was 3.09 (95% CI: 1.54-6.19), the ARR 10.4% (95% CI: 7.8%-12.9%), and the NNT 9.63 (95% CI: 7.70- 12.8). Table 2 shows the adjusted estimators.

## Discussion

This study showed that our population of cancer patients with SARS-CoV2 virus infection and who did not receive the COVID-19 vaccine are at increased risk of hospitalization, mechanical ventilation, and 30-day death from all causes compared with those patients who were vaccinated.

Our data come from an initiative of the Asociacion Colombiana de Hematologia y Oncologia (ACHO) that, faced with the challenge of the COVID-19 pandemic, raised the need to have a national source of information related to the situation of cancer patients and this new infectious pathology given the lack of information worldwide. In response, the ACHOCC-19 national information registry was designed and launched to document the behavior of SARS-CoV-2 infection in cancer patients in Colombia, since the beginning of the pandemic, and favor decision-making in this population. Once some of the COVID-19 vaccines were available, the registry was adjusted to monitor the effect of vaccination on this population.

These results show that the probability of dying within 30 days from all causes is three times higher in the group of unvaccinated cancer patients compared to vaccinated patients (RR: 3.09) and that the risk of dying among cancer patients who received the vaccine compared to those not vaccinated is reduced by 11.5% (ARR: 11.5%). Additionally, and just as relevant, is the fact that it is necessary to vaccinate close to 10 subjects to avoid a fatal outcome in these patients (NNT: 9.63).

Globally, different studies have evaluated the efficacy of vaccines in patients with cancer 19, 20. A systematic review that studied the efficacy of COVID-19 vaccines in immunocompromised patients included 39 studies in cancer patients, all of them focused on documenting seroconversion rates after vaccination 21 18. However, in these patients it is essential to know the impact of vaccines in terms of mortality and hospitalizations. There is limited information about the Latin American population and as far as our knowledge has come, this is the first report made with real-world evidence, which allows corroborating the effectiveness of vaccines against COVID-19 in Colombian patients with solid malignant neoplasms. But on the other hand, worldwide research is being carried out along the same lines. However, to date, the reports are still scarce and mainly focused on summaries that seem to agree on the effectiveness of vaccines in preventing adverse events if the infection is produced. In contrast to the literature, some have concentrated on characterizing the vaccine-receiving cancer population 22, 23, and others have deepened their effectiveness in this vaccinated population 23, 25. Below is a description of the most relevant findings, which, without having studied the same outcomes proposed in our research, are to some extent consistent with our conclusion about the effectiveness of vaccines in cancer populations.

Based on a registry of patients diagnosed with solid or hematological malignancies, Wu et al. 24 compared 29,152 vaccinated patients and the same number of unvaccinated patients to establish the effectiveness of COVID-19 vaccines. The primary outcome was SARS-COV2-confirmed infection. Based on the results obtained, they estimated a relative risk reduction (1-RR) 58% and concluded that vaccination is an effective strategy to prevent COVID-19 infection among cancer patients.

Ben-Aharon and colleagues studied the effectiveness of the BNT162b2 vaccine in a cohort of cancer patients (n=232) compared with a previously healthy control group of health workers (n=261). As the primary outcome, they studied infection with COVID-19 25. After the first dose, seropositivity was reported in 29% of vaccinees compared with 84% of controls, leading the authors to conclude the effectiveness of this vaccine in cancer patients.

Similarly, Thomas and colleagues also evaluated the effectiveness of the BNT162b2 vaccine in cancer patients 25, 26 based on phase III ECC, in which a subgroup analysis evaluated 1647 patients with a previous cancer diagnosis. Compared to placebo, these authors reported a vaccine efficacy of 89.7%.

Regarding the outcome of 30-day mortality from all causes in the population with cancer, it has not been possible to identify other studies that have incorporated it into the analysis. However, we can contrast our findings with the results published by Kornag et al., who conducted a systematic review to evaluate the effectiveness of vaccines against COVID-19 and measured the outcome of all-cause mortality following COVID-19 infection in the general population. Based on the analysis of 7 clinical trials with a combined sample of 168,701 patients, these authors concluded that in the general population, the incidence of mortality in vaccinated patients is more than double that observed in vaccinated patients (RR, 0.41; 95% CI). 0.22 - 0.77) 18. This finding is consistent with that established in the present research, with the additional contribution, which suggest that, in the population with unvaccinated cancer, the risk of dying is threefold than in the vaccinated population.

In our study, different models were used to control possible confounding factors, incorporating variables associated with exposure and outcome. After this analysis, the estimates were adjusted to include age over 60 years, type of health insurance as surrogate of socioeconomic status, metastatic disease (TNM-IV), chemotherapy and breast cancer. Once these factors were adjusted, the estimates of our outcomes of interest made it possible to verify the vaccine's effectiveness in this population group.

In addition, we initially documented in the national registry of patients with cancer and COVID-19 infection ACHOC-C19 during 2020 a high mortality, which was higher among low-income patients. When evaluating the impact of vaccination on mortality according to the type of health care regimen in the low economic level population, it was again documented that this population had a lower frequency of vaccination and a higher mortality rate. This finding may be associated with difficulties in accessing the national health system, which is characterized by its heterogeneity, in addition to the presence of high proportion of patients with uncontrolled comorbidities, that reflects the inadequate medical attention in low-income population and also, problems of misinformation about the indication of vaccination.

The results of this research are not free of bias and have the limitations of observational studies, however its strength lies in being able to demonstrate the behavior of the vaccination strategy for cancer patients in the context of real life. This study is important because it evaluated the effectiveness of the set of vaccines applied in the Colombian context during the first semester of their availability in the country. It also, provided information to identify global risk factors for complications and mortality as well as limitations for access to the health system in our country. The data from this research can be used to generate strategy implementations and improve vaccination coverage that can be extrapolated to other Latin American and low-income countries.

In this cohort we did not evaluate the effectiveness of a particular vaccine; however, we consider that, in subsequent analyses with adequate sample size, it would be pertinent to consider a comparison between the vaccines available for our population.

In conclusion, our data indicate that vaccination against Covid -19 is a strategy whose clinical benefits could outweigh the potential risks, therefore it is recommended to promote it among patients with active cancer and those under follow-up after oncological treatment and to actively reinforce the strategies for disseminating these results to achieve greater vaccine penetration in these patients. At the same time, considering that special measures must be adopted to support the low-income population because they are more vulnerable.

## Figures and Tables

**Table 1:** Baseline characteristics of the study population

Variable	Vaccinated (n= 172)		Non-vaccinated (n= 724)		All (n= 896)	
	n	%	n	%	n	%
Age						
18- 30	1	0.58	23	3.18	24	2.68
31- 40	9	5.23	62	8.56	71	7.92
41- 50	15	8.72	96	13.26	111	12.39
51- 60	37	21.51	182	25.14	219	24.44
61- 70	38	22.09	201	27.76	239	26.67
>70	72	41.86	160	22.10	232	25.89
Sex						
Female	108	62.79	422	58.29	530	59.15
Male	64	37.21	302	41.71	366	40.85
Residence						
Rural	11	6.40	45	6.22	56	6.25
Urban	161	93.60	679	93.78	840	93.75
Socio-economic status						
Low	70	40.70	364	50.28	434	48.44
Medium	76	44.19	255	35.22	331	36.94
High	10	5.81	26	3.59	36	4.02
Not reported	16	9.30	79	10.91	95	10.60
Health regime*						
Subsidized	26	15.12	185	25.55	211	23.55
Contributory	146	84.88	539	74.45	685	76.45
TNM						
I	24	13.95	82	11.33	106	11.83
II	40	23.26	129	17.82	169	18.86
III	58	33.72	220	30.39	278	31.03
IV	40	23.26	241	33.29	281	31.36
Not classified	10	5.81	45	6.22	55	6.14
Not reported	0	0.00	7	0.97	7	0.78
ECOG						
0	64	37.21	165	22.79	229	25.56
1	93	54.07	365	50.41	458	51.12
2	9	5.23	122	16.85	131	14.62
3	6	3.49	55	7.60	61	6.81
4	0	0.00	17	2.35	17	1.90
Type of malignancy						
Breast	68	39.53	153	21.13	221	24.67
Colon-rectum	14	8.14	107	14.78	121	13.50
Prostate	22	12.79	65	8.98	87	9.71
Gastric	11	6.40	53	7.32	64	7.14
Lung	9	5.23	36	4.97	45	5.02
Ovary	7	4.07	30	4.14	37	4.13
Other	41	23.84	280	38.67	321	35.83
Other features						
Obesity	18	10.47	54	7.46	72	8.04
Diabetes	32	18.60	98	13.54	130	14.51
Smoking	5	2.91	17	2.35	22	2.46
Comorbidities>2	21	12.21	74	10.22	95	10.60
Progression	12	6.98	79	10.91	91	10.16
Chemotherapy	39	22.67	163	22.51	202	22.54
Not reported	45	26.16	239	33.01	284	31.70

* In Colombia, the health system has two regimens, the contributory one for people with economic resources to pay and the subsidized one which lower socio-economic level population belongs.

**Table 2:** Multivariate analysis: hospitalization, mechanical ventilation and mortality.

Predictor	Hospitalization		Mechanical ventilation		Mortality 30 days	
	RR (95% CI)	P-value	RR (95% CI)	P-value	RR (95% CI)	P-value
Not vaccinated vs vaccinated	1.36 (1.08- 1.72)	0.009	2.06 (1.01- 4.24)	0.049	3.09 (1.54- 6.19)	0.001
Older than 60 years-old	1.65 (1.39- 1.95)	0.000	2.50 (1.46- 4.27)	0.001	1.87 (1.32- 2.65)	0.000
TNM IV	1.41 (1.21- 1.63)	0.000	1.14 (0.71- 1.81)	0.578	1.90 (1.38- 2.62)	0.000
Subsidized Regime*	1.26 (1.09- 1.46)	0.002	0.91 (0.53- 1.56)	0.744	1.77 (1.29- 2.43)	0.000
Chemotherapy	0.76 (0.62- 0.93)	0.010	0.65 (0.34- 1.21)	0.180	1.00 (0.69- 1.46)	0.959
Breast cancer	0.75 (0.60- 0.95)	0.017	0.58 (0.29- 1.12)	0.110	0.70 (0.43- 1.14)	0.162

* In Colombia, the health system has two regimens, the contributory one for people with economic resources to pay and the subsidized one which lower socio-economic level population belongs.
